# Insomnia Symptoms among African-American Older Adults in Economically Disadvantaged Areas of South Los Angeles

**DOI:** 10.3390/brainsci9110306

**Published:** 2019-11-02

**Authors:** Mohsen Bazargan, Nadia Mian, Sharon Cobb, Roberto Vargas, Shervin Assari

**Affiliations:** 1 Department of Family Medicine, Charles R Drew University of Medicine and Science (CDU), Los Angeles, CA 90059, USA; Mohsenbazargan@cdrewu.edu (M.B.); nadiamian@cdrewu.edu (N.M.); robertovargas@cdrewu.edu (R.V.); 2 Department of Family Medicine, University of California, Los Angeles (UCLA), Los Angeles, CA 90095, USA; 3 School of Nursing, Charles R Drew University of Medicine and Science, Los Angeles, CA 90059, USA; sharoncobb1@cdrewu.edu; 4 Urban Health Institute, Charles R Drew University of Medicine and Science, Los Angeles, CA 90059, USA

**Keywords:** African-Americans, depression, older adults, insomnia, sleep disorder, sleep quality

## Abstract

Background. Although psychosocial and health factors impact insomnia symptoms, less is known about these effects in economically disadvantaged African-American older adults. Aims. This study investigated social and health determinants of insomnia symptoms among economically disadvantaged African-American older adults. Methods. This survey enrolled 398 African-American older adults (age ≥ 65 years) from economically disadvantaged areas of South Los Angeles. Gender, age, educational attainment, financial difficulty, number of chronic diseases, self-rated health, pain intensity, and depression were covariates. Total insomnia, insomnia symptoms, and insomnia impact were our outcomes. Linear regression was applied for data analysis. Results. Based on linear regression, higher financial difficulty (B = 0.48, 95% CI = 0.35–0.61), smoking status (B = 1.64, 95% CI = 0.13–3.16), higher pain intensity (B = 0.39, 95% CI = 0.11–0.67), higher number of chronic diseases (B = 0.34, 95% CI = 0.05–0.64), and more depressive symptoms (B = 0.35, 95% CI = 0.12–0.57) were associated with a higher frequency of insomnia symptoms. Based on a logistic regression model, lower age (B = 0.91, 95% CI = 0.91–1.00) and high financial difficulty (OR = 1.15, 95% CI = 1.08–1.24), pain (OR = 2.08, 95% CI = 1.14–3.80), chronic disease (OR = 1.27, 95% CI = 1.07–1.51) and depression (OR = 2.38, 95% CI = 1.22–4.65) were associated with higher odds of possible clinical insomnia. We also found specific predictors for insomnia symptoms and insomnia impact. Conclusions. Among African-American older adults in economically disadvantaged areas of South Los Angeles, insomnia symptoms co-occur with other economic, physical, and mental health challenges such as financial difficulty, smoking, multimorbidity, pain, and depression. There is a need to address sleep as a component of care of economically disadvantaged African-American older adults who have multiple social and health challenges.

## 1. Background

Insomnia symptoms are highly prevalent and are a significant public health problem. Yet sleep disorders are under-recognized and under-treated issues, especially in older adults. Untreated insomnia is leading to an increase in all-cause health care utilization among older Medicare beneficiaries [1].

Insomnia symptoms refer to a wide range of sleep problems, such as difficulty initiating and/or maintaining sleep, as well as waking up early with an inability to fall back asleep. Insomnia symptoms are commonly accompanied by other symptoms, including but not limited to fatigue, cognitive impairment, memory deficits, lack of concentration, and mood disturbances. About 9–12% of the general population and 25–40% of older adults may be diagnosed with sleep disorders [2]. In the United States, the prevalence of diagnosed insomnia increased by 150% between 2006 and 2013 [1]. Insomnia is more common in women, middle-aged and older adults, and individuals with coexisting medical and psychiatric disorders [2]. 

Empirical data strongly support the powerful influence of insomnia symptoms on cardiometabolic outcomes, and overwhelming evidence suggest that sleep inequalities play an important role in the adverse health disparities in the African-American population [3]. Review studies have uncovered sleep disparities between African-Americans and White Americans, with African-Americans having a higher frequency of insomnia symptoms, increased difficulty in falling asleep, and less deep sleep [4]. Compared to Whites, African-Americans are also more likely to have sleep-disordered breathing. However, data on sleep disorder treatment for African-Americans are limited and inconsistent [5]. 

Moreover, review studies have shown that African-Americans exhibit more risk factors for insomnia when compared to White Americans [5]. Most of what we know about the sleep problems of African-Americans is in younger adults (e.g., middle-aged adults, pregnant women, and adolescents) [5,6,7,8]. Some studies have suggested that sleep differences between Whites and African-Americans are predominantly concentrated in young- and middle-aged adults [5]. However, insomnia symptoms may be more prominent and frequent among older adults [9]. One study found that short sleep duration (<6 h/day) and insomnia symptoms are related to high blood pressure, high fasting blood glucose, and high triglyceride levels among middle-aged and older adults [10]. Aging impacts the frequency of insomnia symptoms, as those who are elderly (age > 65) are more likely to have sleeping issues compared to younger groups [11,12,13]. Yet these studies have mainly focused on cognition, memory deficits, and mood changes, with little research focused on older African-Americans. One 2019 study by Gamaldo et al examined memory and cognition and insomnia symptoms among older African-Americans [14]. The results revealed that insomnia was linked to worse memory and dysfunction. Therefore, mechanisms that contribute to insomnia may differ between African-Americans and Whites [15,16], potentially placing African-Americans at a higher risk of insomnia and sleep problems.

Furthermore, there is a lack of evidence on the evaluation of sleep among African-American older adults and its relationship with health, social, and lifestyle factors. Various factors associated with insomnia symptoms in the African-American population may include socioeconomic status (SES) [17], discrimination [18], chronic pain [19], mental illness [6], self-rated health [20], and chronic disease [21]. In addition, studies focused on older adults have found that smoking and alcohol use directly affect the duration and quality of sleep, yet this is under-examined within the older African-American population [22,23]. Altogether, these factors have not been well-studied among the older age cohort of African-Americans in relation to the frequency of insomnia symptoms, which may be linked to adverse health outcomes in this group. Even though racial disparities in sleep have been clearly documented, there is a lack of knowledge on whether specific factors (such as socioeconomic status, chronic diseases, pain, depression, and self-rated health) may explain sleep outcomes among older African-Americans. It is imperative that these factors be investigated to understand how insomnia symptoms may be influenced by personal and social determinants among African-American older adults [9,24]. There is a particular dearth of knowledge on the social and health determinants of the frequency of insomnia symptoms in economically disadvantaged African-American older adults.

### Aims

This study aimed to investigate psychosocial and health correlates of the frequency of insomnia symptoms among economically disadvantaged African-American older adults. Based on a literature review, we hypothesized that age [5], gender [25], SES [15,26], chronic diseases [15], self-rated health [27,28], pain intensity [29], and depression [30] would be associated with insomnia symptoms in this population. We also explored predictors of insomnia symptoms and insomnia impact, as two factors of the Insomnia Symptoms Index (ISI).

## 2. Materials and Methods

### 2.1. Design & Setting

This cross-sectional study was a survey conducted in South Los Angeles between 2015 and 2018. More details of methodology and sampling are available elsewhere [31,32,33,34,35,36,37,38,39]. 

### 2.2. Participants

African-American older adults were selected from low SES areas in South Los Angeles, CA. This urban region, known as Service Planning Area 6 (SPA 6), is one of the most economically disadvantaged urban areas in Los Angeles, due to lower life expectancy rates, reduced access to employment and healthcare resources, and lower household income [40]. Among older African-American adults in this area, over 35% rate their health as fair or poor, and an estimated 30% have a high school diploma [40].

Using a convenience sampling, African-American older adults were enrolled if they were African-American / Black, 65+ years, lived in SPA 6, and could understand consent and then complete an interview in English. Institutionalized participants were not enrolled in the study. Other exclusion criteria included participation in any other clinical trials that could interfere with the study constructs. The total sample was 398 economically disadvantaged African-American older adults who were 65+ years old.

### 2.3. Data Collection

The data were collected via structured face-to-face interviews. The information gathered during the interviews included demographic factors (age and gender), SES (educational attainment and financial difficulty), and health (chronic diseases, self-rated health, pain intensity, and depression).

### 2.4. Measurements

#### 2.4.1. Independent Variables

*Demographic Characteristics*. Age and gender were covariates. Age was treated as an interval variable. Gender was dichotomous: 1 male, 0 female. 

*Socioeconomic status (SES)*. Education attainment and financial difficulty were the SES variables. Educational attainment was a continuous variable that reflected years of schooling. Financial difficulty was measured using a three-item measure. Our self-reported perceived measure of financial distress was consistent with Pearlin’s conceptualization of financial difficulties of low SES individuals. The items measured lack of cash to afford clothing, food, and difficulty with paying bills. Response to these items ranged from 1 for “never” to 5 for “always.” A higher score reflected more financial difficulty. Cronbach’s alpha was 0.92.

*Number of Chronic Diseases*. Participants reported the presence of the following chronic diseases: chronic obstructive pulmonary disease, hypertension (i.e., high blood pressure), heart disease (e.g., heart attack), thyroid disorder, diabetes mellitus, lipid disorder, cancer, osteoarthritis, asthma, rheumatoid arthritis, and gastrointestinal disease. Participants were asked if they were ever told by a physician that they have the above conditions. We treated the number of chronic diseases as a continuous variable with a higher score indicating more chronic diseases. 

*Self-Rated Health*. Self-rated health status was measured using a single item regarding participants’ overall health. Responses ranged from 1 for “excellent” to 5 for “poor.” Having a poor self-rated health is predictive of all-cause mortality in both community and clinical settings [41].

*Depressive Symptoms*. The measure of depression was the 15-item Geriatric Depression Scale - Short Form (GDS-SF) [42,43,44,45,46]. Each item was answered with a “yes” (1) or a “no” (0). The total score ranged between 0 and 15, with a higher score indicating more depressive symptoms. The GDS-SF has excellent reliability and validity and is frequently used to identify older adults who may at be risk of, or managing, depression. For our logistic regression, we entered significant depressive symptoms (score ≥ 5) as a predictor.

*Pain Intensity*. We measured pain intensity using four subscales of the McGill Pain Questionnaire - Short Form 2 (MPQ-SF-2) [47]. Participants provided responses to 22 pain items that assessed the types of pain they have experienced in the past 7 days, including the extent and intensity. Each item was evaluated on a 11-point numeric rating scale from 0 (none) to 10 (worst possible). The subscales of the MPQ-SF-2 include: (a) Continuity (throbbing, cramping, gnawing, aching, heavy, and tender pain), (b) intermittence (shooting, stabbing, sharp, splitting, electric-shock, and piercing pain), (c) neuropathic nature (hot-burning, cold-freezing, itching, tingling or “pins and needles,” light touch, and numbness pain), and (d) affective domain (tiring-exhausting, sickening, fearful, and punishing-cruel pain). A total pain score was calculated after averaging all responses [47]. A higher score is indicative of greater intense chronic pain. For our logistic regression, we entered the highest quartile of the pain intensity as a predictor.

*Alcohol and Smoking*. Participants were asked about their smoking (cigarette) and alcohol use. Respondents were asked: “How would you describe your cigarette smoking habits?” and “Do you drink alcohol?” Response items to the first question were: (1) never smoked, (2) previously smoked, and (3) current smoker. The answer to the second question included both yes and no. These two variables were operationalized as dichotomous variables.

#### 2.4.2. Outcome Variable

*Insomnia Symptoms Frequency*. Our main dependent variable was the frequency of insomnia symptoms, measured using the 7-item Insomnia Severity Index (ISI) [48,49]. The ISI is the recommended measure for all insomnia studies [49,50,51]. The seven items were: (1) “Please rate the current (i.e., within the last 2 weeks) severity of difficulty falling asleep”, (2) “Please rate the current (i.e., within the last 2 weeks) severity of difficulty staying asleep”, (3) “Please rate the current (i.e., within the last 2 weeks) severity of problem waking up too early”, (4) “How satisfied/dissatisfied are you with your current sleep pattern?,” (5) “To what extent do you consider your sleep to interfere with your daily functioning”, (6) “How noticeable to others do you think your sleeping problem is in terms of impairing the quality of your life?”, and (7) “How worried/distressed are you about your current problem?” Each item was on a Likert scale with possible answers from 0 to 4. We calculated a total “*Insomnia Symptoms Frequency*” score ranging from 0 to 28, with a higher score reflecting more insomnia symptoms. 

For our logistic regression, we classified our insomnia score as follows: (a) 0–14, no clinically significant insomnia, (b) 15–28, possible clinical insomnia (moderate to severe insomnia). Very few individuals had severe and moderate insomnia, thus we combined all levels to have a robust outcome. Research shows that the ISI has adequate validity and internal consistency (reliability) to evaluate sleep difficulties [48,49].

Many scholars have simply calculated the insomnia total score, assuming that ISI only has one single factor [49,50,51]. These researchers have used ISI without defining any sub-scales [49,50,51]. Recent research, however, suggests that ISI can be seen as a two- or three- factor solutions [52,53]. In our sample, which was composed of African-American older adults in economically challenged areas of south Los Angeles, we found supporting evidence for a two-factor model. While the first four items were used to determine the insomnia symptom score, the last three items were used to determine the insomnia impact [52,53]. Thus, we defined three scores for our ISI outcomes: Total score, Factor 1 (insomnia symptoms), and Factor 2 (insomnia impact) [52,53].

### 2.5. Data Analysis

Statistical analysis was performed using SPSS 22.0 (IBM Corporation, Armonk, NY, USA). To describe the sample, we reported mean, standard deviation (SD), and frequency (%). Pearson’s correlation test was utilized to test the bivariate correlations between the variables. We performed separate multivariable models to test predictors of insomnia symptom frequency (as a continuous measure), as well as possible insomnia as a categorical variable. For multivariable analysis with possible insomnia as a categorical outcome, we applied a logistic regression model. For multivariable analysis with insomnia symptom frequency as a continuous measure, we applied three linear regression models. Model 1 has the total ISI score as the outcome. Model 2 and Model 3 have ISI Factor one (insomnia symptoms) and ISI Factor 2 (insomnia impact). In all models, demographic factors (age and gender), SES (educational attainment and financial difficulty), and health (self-rated health, chronic disease, and depression) were the independent variables. For our linear regression, which functions better with continuous measures, we entered pain intensity and depressive symptoms as continuous predictors (raw scores). For our binary logistic regression, which functions best with categorical predictors, we entered threshold (4th highest quartile) of pain intensity and depressive symptoms as predictors. From our linear regression model, we reported B (coefficient), standard error (SE), 95% confidence interval (CI), and p values. From our logistic regression model, we reported odds ratio (OR), SE, and p values.

### 2.6. Institutional Review Board (IRB)

All the participants provided a signed informed consent. The protocol of the current investigation was approved by the IRB at the Charles R. Drew University of Medicine and Science (CDU University, Approval Number: CDU IRB# 14-12-2450-05).

## 3. Results

### 3.1. Descriptive Statistics

From the total number of 398 participants who entered this analysis, most were African-American women (*n* = 258; 64.8%) (See Table 1). The average age of the participants was 73 (SD = 7) years. The mean number of insomnia symptoms (ISI score) was 4.1 (SD = 5.4). 

### 3.2. Various Insomnia Symptoms

Various insomnia symptoms were reported by 17% to 31% of participants, with variation in the symptom type. The most common symptom was difficulty with sleep maintenance (31%), followed by difficulty with sleep initiation (26%) and waking up too early (25%). A total of 26% were dissatisfied with their sleep quality, and 22% and 17% of participants reported that their sleep problem was interfering with their daily functioning and quality of life, respectively. In addition, 18% were worried/distressed about their current sleep problem.

### 3.3. Bivariate Correlations

Table 2 shows a correlation matrix between all the study variables. Younger age (r = −0.15), female gender (r = −0.10), higher financial difficulty (r = −0.40), higher number of chronic diseases (r = −0.27), worse self-rated health (r = −0.21), higher pain intensity (r = 0.35), and higher depressive symptoms (r = −0.31) were correlated with higher frequency of insomnia symptoms. However, educational attainment, smoking, and drinking were not correlated with frequency of insomnia symptoms in the bivariate test. 

### 3.4. Multivariable Logistic Regression Model

As Table 3 shows, high age (B = 0.91, 95% CI = 0.91–1.00) was protective against odds of possible clinical insomnia. High financial difficulty (OR = 1.15, 95% CI = 1.08–1.24), pain (OR = 2.08, 95% CI = 1.14–3.80), chronic disease (OR = 1.27, 95% CI = 1.07–1.51) and depression (OR = 2.38, 95% CI = 1.22–4.65) were associated with higher odds of possible clinical insomnia. Gender, education, smoking, drinking, and self–rated health did not predict possible clinical insomnia (*p* > 0.05).

### 3.5. Multivariable Linear Regression Models

As Table 4 shows, higher financial difficulty (B = 0.48, 95% CI = 0.35–0.61), smoking status (B = 1.64, 95% CI = 0.13–3.16), higher pain intensity (B = 0.39, 95% CI = 0.11–0.67), higher number of chronic diseases (B = 0.34, 95% CI = 0.05–0.64), and more depressive symptoms (B = 0.35, 95% CI = 0.12–0.57) were associated with a higher frequency of insomnia symptoms. Age, gender, drinking, and self–rated health did not predict frequency of insomnia symptoms (*p* > 0.05).

Table 4 also shows predictors of Factor 1 (insomnia symptoms) and Factor 2 (insomnia impact). While chronic diseases (n) (B = 0.07, 95% CI = 0.01–0.13, *p* = 0.018) and pain intensity (B = 0.27, 95% CI = 0.03–0.51, *p* = 0.029) were associated with Factor 1 (insomnia symptoms) not Factor 2 (insomnia impact), depressive symptoms were correlated with Factor 2 (insomnia impact) (B = 0.59, 95% CI = 0.33–0.85, *p* < 0.001) but not Factor 1 (insomnia symptoms). Financial difficulty was associated with Factor 1 (insomnia symptoms) (B = 0.04, 95% CI = 0.01–0.07, *p* = 0.004) and Factor 2 (insomnia symptoms) (B = 0.09, 95% CI = 0.06–0.11, *p* < 0.001). Drinking was also associated with Factor 1 (insomnia symptoms) (B = 0.26, 95% CI = 0.04–0.48, *p* = 0.022) but not Factor 2 (insomnia impact).

## 4. Discussion

In a sample of African–American older adults in an economically disadvantaged area of South Los Angeles, this study showed that financial difficulty, smoking, pain intensity, depression, and a higher number of chronic diseases were predictive of insomnia symptom frequency, and that age, financial difficulty, pain intensity, chronic disease, and depression were associated with higher odds of possible clinical insomnia. We also found different characteristics predicting insomnia symptoms and insomnia impact. While chronic diseases, pain intensity, and drinking were associated with insomnia symptoms, depressive symptoms were correlated with insomnia impact, and financial difficulty was associated with both insomnia symptoms and insomnia impact. 

Our findings were consistent with reviews and empirical studies on insomnia symptoms in African–Americans; however, such research has been mainly conducted in young and middle–aged individuals [6]. Research shows that pain intensity, depression, and chronic morbidity are associated with insomnia in African-Americans [6]. This study extends the literature by examining factors associated with insomnia in African–American older adults. As previous research has shown, SES [54], chronic diseases [55], pain [56], depression [55,57,58], and self-rated health (SRH) [59] are all risk factors for insomnia among minority groups, including African Americans. Low SES [60], chronic diseases [60], pain [61], depression [61], and poor self–rated health are among various aspects of health disparities in ethnic minorities, including African–Americans. Various social, medical, and mental risk factors of insomnia symptoms tend to covary, and require further studies among African–American older adults. Social, behavioral, and medical mechanisms may also contribute to sleep disparities between African-Americans and Whites [15,16]. The current findings among older African–American adults extend the health disparities literature as they reveal that these risk factors persist across age cohorts. We need further research to understand the full extent of the factors associated with insomnia across age groups of African-Americans [62]. 

Depression was associated with insomnia symptoms in our sample. Similar results are shown for African-Americans who were younger and had better SES [55]. Similarly, chronic diseases are shown to be associated with insomnia regardless of race, age, and SES [55]. 

In our study, age was associated with possible clinical insomnia but not frequency of insomnia symptoms. In another study among African–Americans, age was a risk factor for sleep problems [63]. In some other studies in African-Americans and Whites [64], education has some protective effects against insomnia, but such an effect was not found in our sample.

Insomnia is correlated with various social, physical, and mental health problems [18]. Multiple studies have shown that individuals with insomnia tend to have financial difficulty, multiple chronic diseases, and depression [20,65,66]. Thus, individuals with insomnia require holistic and integrated care that addresses physical and mental health as well as social determinants of health [10,21]. African–American older adults with multiple chronic diseases or depression, particularly those who have financial difficulty, should be screened for insomnia [67]. Previous studies have documented the impact of socioeconomic factors and health indicators on insomnia symptoms among African-Americans [68]. Clinicians should be aware of the cumulative effects of financial difficulty, depression, pain, and multimorbidity on insomnia symptoms for African–American older adults who are financially disadvantaged.

As low SES, depression, multimorbidity, pain, and insomnia tend to co–occur, there is a need to address the social, mental, and physical health of low SES African–American individuals simultaneously as part of a comprehensive effort to promote the health and well–being of African–American communities [4,17]. Care for insomnia should be delivered to African–American older adults who seek care for multimorbidity, depression, and pain, to improve health outcomes. The solution seems to be combined comprehensive programs and interventions that go beyond one aspect of a patient’s needs and that address multiple needs across domains [69]. 

As different characteristics predict insomnia symptoms and insomnia impact, prevention of insomnia symptoms and insomnia impact may have different requirements. While chronic diseases, pain intensity, and drinking are linked to insomnia symptoms, depressive symptoms seem to be relevant to insomnia impact. Interestingly, financial difficulty is associated with both insomnia symptoms and insomnia impact. Thus, some interventions may enhance one but not the other aspect of insomnia. 

Our results are specific to an urban sample of African–American older adults who live in an economically challenged area. The results should be cautiously compared with the literature on other demographic groups, across locations, racial and ethnic groups, and SES levels [15,16,70,71]. Our findings may not be relevant to Whites, younger African–American adults, and even African–American older adults with higher SES. 

We gain some additional insight by comparing our bivariate correlations (shown in Table 2) with similar associations reported in Whites or younger African-Americans reported in the literature [72]. Educational attainment was not correlated with insomnia symptoms in bivariate or multivariable tests. Similarly, health behaviors such as smoking and drinking were not correlated with insomnia in bivariate tests. These patterns in our sample stand in contrast to others reported in the literature. Although education is shown to be associated with lower insomnia symptoms in Whites and younger African–Americans, education shows smaller protective effects in populations who live in economically constrained contexts, a pattern also known as minorities’ diminished returns (MDRs). MDRs refer to attenuated correlations and associations of SES indicators for African–Americans. The degree of the association between depressive symptoms and insomnia symptoms also seems to be lower than what was previously found for Whites and younger African-Americans [73,74,75,76]. 

The findings of this study can inform public health policies and programs as they relate to sleep disparities in low–income African–American populations [77]. Programs that wish to consider insomnia should also screen for comorbid pain, depression, and CMCs [78,79]. Untreated pain among African–American older adults is another major factor that contributes to insomnia among this segment of our population. Reassuringly, all of these factors are modifiable. 

Detecting and treatment of sleep problems may be a salient part of improving the overall well–being of African–American older adults in economically challenged communities. Because insomnia was associated with social, medical, and mental adversities in African–American older adults, future research should test whether insomnia mediates some of the health disparities in this population. 

The elderly (like the very young) tend to sleep in a polyphasic (i.e., multiple sleep episodes per day) pattern rather than the monophasic pattern characteristic of younger adults. This pattern in the elderly makes assessment of insomnia difficult. This is important considering the average age was 74 years in the study cohort.

## 5. Limitations

Every study has some limitations. First, cross–sectional studies cannot make causal inferences. Thus, our results only indicate association, not causation. Second, some omitted confounders may have biased the results. For example, a history of psychiatric problems, somatization, and other conditions that closely overlap with insomnia were not included in this study. Additionally, household income, a strong SES indicator, was not measured. Although we did not have data on income, we used additional measures of SES, such as financial difficulty. Similarly, we did not examine medication, diet, and exercise. Certain medications impact individuals’ capacity to initiate or maintain sleep. Drinks and foods that contain stimulants (e.g., coffee, tea, and chocolates) may also interfere with sleep. Finally, this study was on a convenience sample, not a truly randomized sample. Therefore, the results cannot be generalized to all African–Americans. Future research is needed to replicate these findings in nationally representative samples. Despite these limitations, this study contributes to the existing knowledge on insomnia among African–American older adults in economically disadvantaged urban settings.

## 6. Conclusions

For African–American older adults who live in economically disadvantaged areas, insomnia is linked with financial difficulty, multiple chronic diseases, depression, and pain. Given the existing overlap between these social and health conditions, interventions that target insomnia in African–American older adults should simultaneously address financial needs, multimorbidity, depression, and pain. 

## Figures and Tables

**Table 1:** Descriptive characteristics.

Characteristic	All	
	***n***	**%**
Gender		
Male	140	35.2
Female	258	64.8
Smoking (Any)		
No	53	13.3
Yes	345	86.7
Drinking (Any)		
No	289	72.6
Yes	109	27.4
	**Mean**	**SD**
Age (Years)	73.5	7.0
Educational Attainment	12.8	2.3
Financial Difficulty	6.7	3.7
Chronic Diseases	3.5	1.8
Self-Rated Health (1–5)	3.0	1.0
Pain Intensity (Score)	1.6	2.0
Depressive Symptoms (Score)	1.7	2.3
Insomnia Symptoms (Score)	4.1	5.4

**Table 2:** Bivariate correlations.

	1	2	3	4	5	6	7	8	9	10	11
1 Age	1	−0.05	−0.19 ^**^	−0.14 ^**^	0.27 ^**^	−0.10 ^*^	0.00	−0.22 ^**^	−0.16 ^**^	−0.13 ^**^	−0.15 ^**^
2 Gender (Male)		1	−0.10 ^*^	−0.03	−0.27 ^**^	0.04	−0.10	−0.06	−0.09	−0.03	−0.10 ^*^
3 Educational Attainment			1	−0.07	0.03	0.08	−0.08	0.01	−0.00	0.00	0.02
4 Financial Difficulty				1	−0.15 ^**^	0.11 ^*^	0.12 ^*^	0.14 ^**^	0.21 ^**^	0.17 ^**^	0.40 ^**^
5 Smoking (Any)					1	−0.24 ^**^	0.10 ^*^	−0.13 ^*^	−0.01	−0.14 ^**^	0.01
6 Drinking (Any)						1	−0.10 ^*^	0.05	0.02	0.07	0.09
7 Chronic Diseases (n)							1	0.23 ^**^	0.45 ^**^	0.29 ^**^	0.27 ^**^
8 Self–Rated Health (1–5)								1	0.34 ^**^	0.28 ^**^	0.21 ^**^
9 Pain Intensity (Score)									1	0.39 ^**^	0.35 ^**^
10 Depressive Symptoms (Score)										1	0.31 ^**^
11 Frequency of insomnia Symptoms											1

* *p* < 0.05; ** *p* < 0.001.

**Table 3:** Logistic regression on predictors of odds of possible clinical insomnia

	OR	95% CI	*p*
Age (Years)	0.95	0.91–1.00	0.041
Gender (Male)	0.68	0.36–1.27	0.225
Educational Attainment (Years)	1.02	0.89–1.16	0.787
Financial Difficulty (Score)	1.15	1.08–1.24	0.000
Smoking (Any)	0.54	0.22–1.36	0.192
Drinking (Any)	1.79	0.97–3.28	0.062
Self–Rated Health (1–5)	1.11	0.82–1.50	0.486
Chronic Disease (n)	1.27	1.07–1.51	0.006
Pain Intensity (Threshold)	2.08	1.14–3.80	0.017
Depression (Threshold)	2.38	1.22–4.65	0.011
Intercept	0.52		0.764

OR: Odds Ratio.

**Table 4:** Linear regressions on predictors of insomnia symptoms.

	Beta	SE	B	95% CI for B	*p*
**Total Score**					
Age (Years)	0.04	−0.08	−0.06	−0.13–0.01	0.102
Gender (Male)	0.51	−0.03	−0.39	−1.40–0.61	0.444
Educational Attainment (Years)	0.11	0.04	0.09	−0.12–0.30	0.397
Financial Difficulty (Score)	0.07	0.33	0.48	0.35–0.61	<0.001
Smoking (Any)	0.77	0.10	1.64	0.13–3.16	0.034
Drinking (Any)	0.54	0.07	0.89	−0.18–1.95	0.103
Self–Rated Health (1–5)	0.26	0.04	0.20	−0.31–0.70	0.451
Chronic Disease (n)	0.15	0.11	0.34	0.05–0.64	0.022
Pain (Score)	0.14	0.14	0.39	0.11–0.67	0.006
Depressive Symptoms (Score)	0.11	0.15	0.35	0.12–0.57	0.003
Intercept	3.50		−0.47	−7.35–6.41	0.893
**Factor 1 (Symptoms)**					
Age (Years)	0.00	0.01	−0.02	−0.02–0.01	0.704
Gender (Male)	−0.19	0.11	−0.09	−0.40–0.01	0.068
Educational Attainment (Years)	0.03	0.02	0.07	−0.01–0.07	0.164
Financial Difficulty (Score)	0.04	0.01	0.15	0.01–0.07	0.004
Smoking (Any)	−0.18	0.16	−0.06	−0.49–0.13	0.257
Drinking (Any)	0.26	0.11	0.12	0.04–0.48	0.022
Self–Rated Health (1–5)	0.02	0.05	0.02	−0.08–0.13	0.635
Chronic Disease (n)	0.07	0.03	0.13	0.01–0.13	0.018
Pain (Score)	0.27	0.12	0.12	0.03–0.51	0.029
Depressive Symptoms (Score)	−0.04	0.14	−0.02	−0.32–0.24	0.761
Intercept	−0.83	0.73		−2.27–0.61	0.257
**Factor 2 (Impact)**					
Age (Years)	−0.01	0.01	−0.09	−0.03–0.00	0.072
Gender (Male)	0.11	0.10	0.05	−0.08–0.30	0.272
Educational Attainment (Years)	−0.01	0.02	−0.02	−0.05–0.03	0.611
Financial Difficulty (Score)	0.09	0.01	0.32	0.06–0.11	<0.001
Smoking (Any)	−0.26	0.15	−0.09	−0.55–0.03	0.078
Drinking (Any)	−0.05	0.10	−0.02	−0.26–0.15	0.607
Self–Rated Health (1–5)	0.06	0.05	0.06	−0.04–0.15	0.244
Chronic Disease (n)	0.03	0.03	0.05	−0.03–0.08	0.332
Pain (Score)	0.18	0.11	0.08	−0.04–0.40	0.117
Depressive Symptoms (Score)	0.59	0.13	0.21	0.33–0.85	<0.001
Intercept	0.08	0.68		−1.25–1.42	0.902
